# Disinfection By-products and Bladder Cancer: Common Genetic Variants May Confer Increased Risk

**DOI:** 10.1289/ehp.118-a491a

**Published:** 2010-11

**Authors:** Kris S. Freeman

**Affiliations:** **Kris S. Freeman** has written for *Encarta* encyclopedia, NIH, ABCNews.com, and the National Park Service. Her research on the credibility of online health information appeared in the June 2009 *IEEE Transactions on Professional Communication*

Disinfection of the water supply is an important and cost-effective tool to reduce morbidity and mortality from a wide range of infectious diseases. However, the chemicals used to treat water also can produce potentially toxic compounds known as disinfection by-products (DBPs). A new study shows strong associations between DBP exposure and bladder cancer among individuals who carry inherited variants in three genes (*GSTT1*, *GSTZ1*, and *CYP2E1*) that code for key enzymes that metabolize DBPs **[***EHP* **118(11):1545–1550; Cantor et al.]**.

DBPs form when disinfectants (such as chlorine) react with organic matter that collects in water (such as algae or humic acids from decayed leaves). Most DBP exposure is due to ingestion of drinking water, although some DBPs can be inhaled or absorbed through the skin during bathing, showering, or swimming in a pool. Laboratory studies show that many DBPs are mutagenic or carcinogenic, but epidemiologic studies to date have revealed only a modest association between DBP exposure and cancer in humans.

In the present study, 595 men and 85 women newly diagnosed with bladder cancer were recruited from 18 hospitals in Spain and matched with controls (622 men and 92 women) who had been hospitalized with conditions thought to be unrelated to bladder cancer. The authors estimated DBP exposure since age 15 years by linking participants’ residential histories with documented and estimated levels of trihalomethanes (THMs)—a DBP often used as a marker for total DBP exposure—in municipal water systems. Participants were genotyped for variations in *GSTT1*, *GSTZ1*, and *CYP2E1*.

Across the study population cancer risk nearly doubled between the highest and lowest levels of DBP exposure, and the association with DBP exposure was even stronger among participants who carried one of three variant genotypes. Smokers also had a higher risk (smoking is the most significant known risk factor for bladder cancer).

One of the genotypes appeared to increase the association between DBPs and bladder cancer codes for the active form of the enzyme glutathione transferase theta-1 (GSTT1), which metabolizes brominated THMs to mutagens. Another increases the activity of cytochrome P450 2E1 (CYP2E1), which catalyzes the primary oxidation of THMs. There is evidence the third genotype may reduce the activity of glutathione transferase zeta-1 (GSTZ1), which transforms haloacetic acids, another type of DBP, to less toxic compounds.

Among individuals who carried both of the *GSTT1* and *GSTZ1* genotypes noted above (28% of study participants), those with the highest DBP exposure were at a 1.5 times increased risk of bladder cancer compared with carriers with the lowest DBP exposure. These genotypes are relatively common, occurring jointly in more than 20% of the controls in the study population. The findings from this study therefore may have significant public health implications for cancer prevention.

